# Global Publication Pattern in Ankle Arthroscopy: A Scopus®-Indexed Bibliometric Visualization Analysis

**DOI:** 10.7759/cureus.82294

**Published:** 2025-04-15

**Authors:** Tauseef Ahmad, Kaylem M Feeney

**Affiliations:** 1 School of Public Health, Zhejiang University, Hangzhou, CHN; 2 Orthopaedics, University Hospital Galway, Galway, IRL

**Keywords:** analysis, ankle and foot, arthroscopy, bibliometrics, network collaboration

## Abstract

Ankle arthroscopy has become a common procedure performed by foot and ankle surgeons worldwide in the evaluation and management of ankle pathology. This study aimed to assess and characterize the global trends and network collaboration in ankle arthroscopy research. A retrospective bibliometric study was conducted using predefined keywords. The data utilized in this study were extracted from the Scopus® database. The data were imported into Bibliometrix, an R-tool for prerequisite analysis and network collaboration mapping. A total of 938 peer-reviewed publications (original articles = 778, review articles = 160) were analyzed and characterized. The included articles were published in English between 1973 and 2021. These articles were published in 175 journals and were authored by 2,708 authors. The collaboration index among the authors was 3.08. The most frequent year of publication was 2020 (n = 84). The overall annual scientific growth rate was 10.41%. The top-ranked core journal in ankle arthroscopy was “Foot and Ankle International” (n = 121). The most frequently appeared affiliations were North District Hospital (n = 50), Harvard Medical School (n = 46), and University of Amsterdam (n = 44). The largest number of publications was produced in the United States (n = 376), followed by the United Kingdom (n = 107) and Japan (n = 68). The most studied trend topics were arthroscopy, human, male, adult, and female. The largest portion of the included publications was produced in developed countries, although it is possible that the exclusion of studies published in a language other than English may have influenced these results. The United Kingdom had the strongest collaboration with Italy, France, and Spain. However, the United States had the strongest collaboration with Italy, Spain, and the United Kingdom.

## Introduction and background

Ankle arthroscopy is a diagnostic and therapeutic procedure, which was first performed on a patient in 1939, with the first publication of a series of patients undergoing ankle arthroscopy published in 1972 [1]. Since then, ankle arthroscopy has become a common procedure performed by foot and ankle surgeons worldwide in the evaluation and management of ankle pathology. Ankle arthroscopy is a minimally invasive diagnostic procedure that can also be used in the management of both simple and complex ankle pathology. Benefits of ankle arthroscopy over traditional open surgery include faster recovery time, reduced risk of infection, superior operative view, better cosmesis due to smaller scars, less wound problems, the ability to evaluate the entire ankle joint in real time, and the ability to both diagnose and treat pathology at the same time. Indications for ankle arthroscopy include but are not limited to the management of osteochondral lesions, ankle impingement, removal of loose bodies, synovitis, and ankle arthrodesis [2].

A bibliometric analysis is a quantitative study of scientific publications. The aim of a bibliometric analysis is to identify the most important research articles in a field of study and to map and identify trends in a research field. Bibliometric analyses are important as they serve as a way of highlighting global research output on a topic and identifying key developments in that topic. A bibliometric analysis also facilitates the development of a visualization analysis of research on a topic. Several bibliometric analyses have been performed in the field of foot and ankle surgery [3-8]. However, to the best of our knowledge, none of these studies relate specifically to ankle arthroscopy. Therefore, the aim of this study was to perform a bibliometric analysis to identify global publication patterns in the study of ankle arthroscopy, and this is the first bibliometric analysis to be carried out on this topic.

## Review

Materials and methods

This bibliometric analysis was conducted in line with previously published studies [9-10]. All the relevant scientific literature on ankle arthroscopy was retrieved from the database Scopus®. The following keywords were used to search the title and abstract of potential studies: "ankle arthroscopy" OR "ankle arthroscopic surgery". These keywords were chosen to identify the most relevant studies to the topic of interest. The initial search yielded a total of 1,188 documents. The search was then limited to document types (article and review) and publishing language (English). After filtering the initial search by document type and limiting the publishing language to English, a total of 972 articles remained. These 972 articles were screened by the two authors of this study. Articles published in the year 2022 and articles that were in press were excluded from the final analysis. After screening and the application of filters, a total of 938 articles were included in the final analysis. Articles were excluded if they were not published in the English language due to resource limitations. Articles that were not peer-reviewed original or review articles were also excluded. 

**Figure 1:**
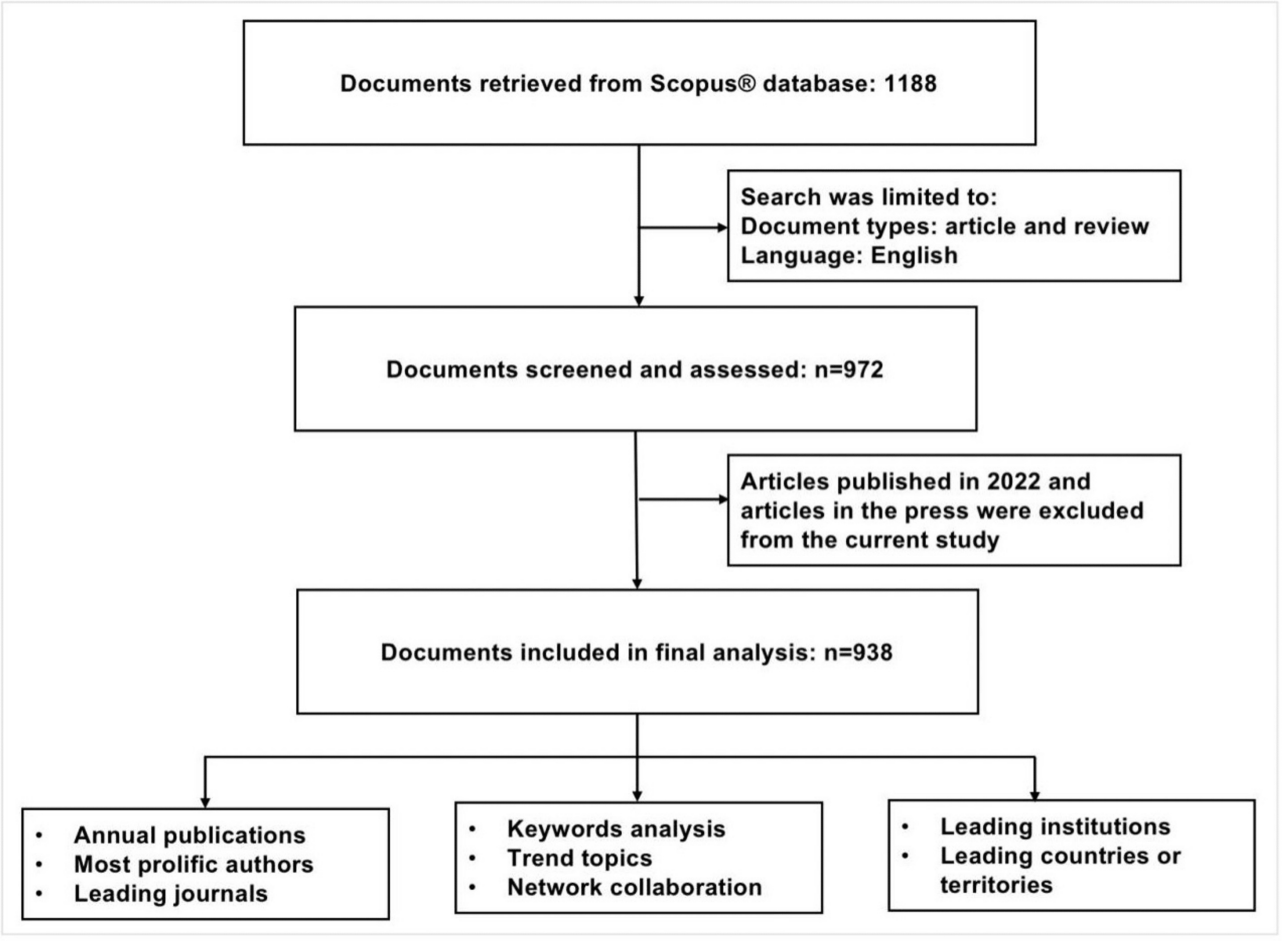
Flow chart of the included and analyzed documents

The 938 screened publications dataset was downloaded in comma-separated values format. A formal analysis of the 938 included studies was performed by the first author of this study. A flow chart of the studies included in the final analysis is shown in Figure 1. The following parameters were extracted from the data: title, year of publication, names of authors, journal name, keywords, institution, and country of origin. The data was imported to Bibliometrix, an R-tool for pre-requisite analysis. The journal impact factor (IF) was obtained from the Journal Citation Reports released by Clarivate Analytics in June 2022. The characteristics of the included articles are discussed in the results section. In this study, no animal or human subjects were directly involved. The data utilized in the study was extracted from a publicly available database (Scopus®). Therefore, no ethical approval or consent form for publication was required.

Results

In this study, a total of 938 publications (article = 778, review = 160) were analyzed and characterized, as shown in Table 1. The included articles were published in English between 1973 and 2021. These articles were published in 175 journals and were authored by 2,708 authors. The collaboration index among the authors was found to be 3.08. 

**Table 1:** Study characteristics

Description	Results
Time span of the included studies	1973-2021
Journals	175
Documents	938
Average years from publication	10
Average citations per document	20.05
Average citations per year per document	1.766
References	1
Document types	
Article	778
Review	160
Document contents	
Keywords Plus (ID)	3403
Author's keywords (DE)	1327
Authors	
Authors	2708
Author appearances	3754
Authors of single-authored documents	51
Authors of multi-authored documents	2657
Authors collaboration	
Single-authored documents	75
Documents per author	0.346
Authors per document	2.89
Co-authors per documents	4
Collaboration index	3.08

Annual Scientific Production

**Figure 2:**
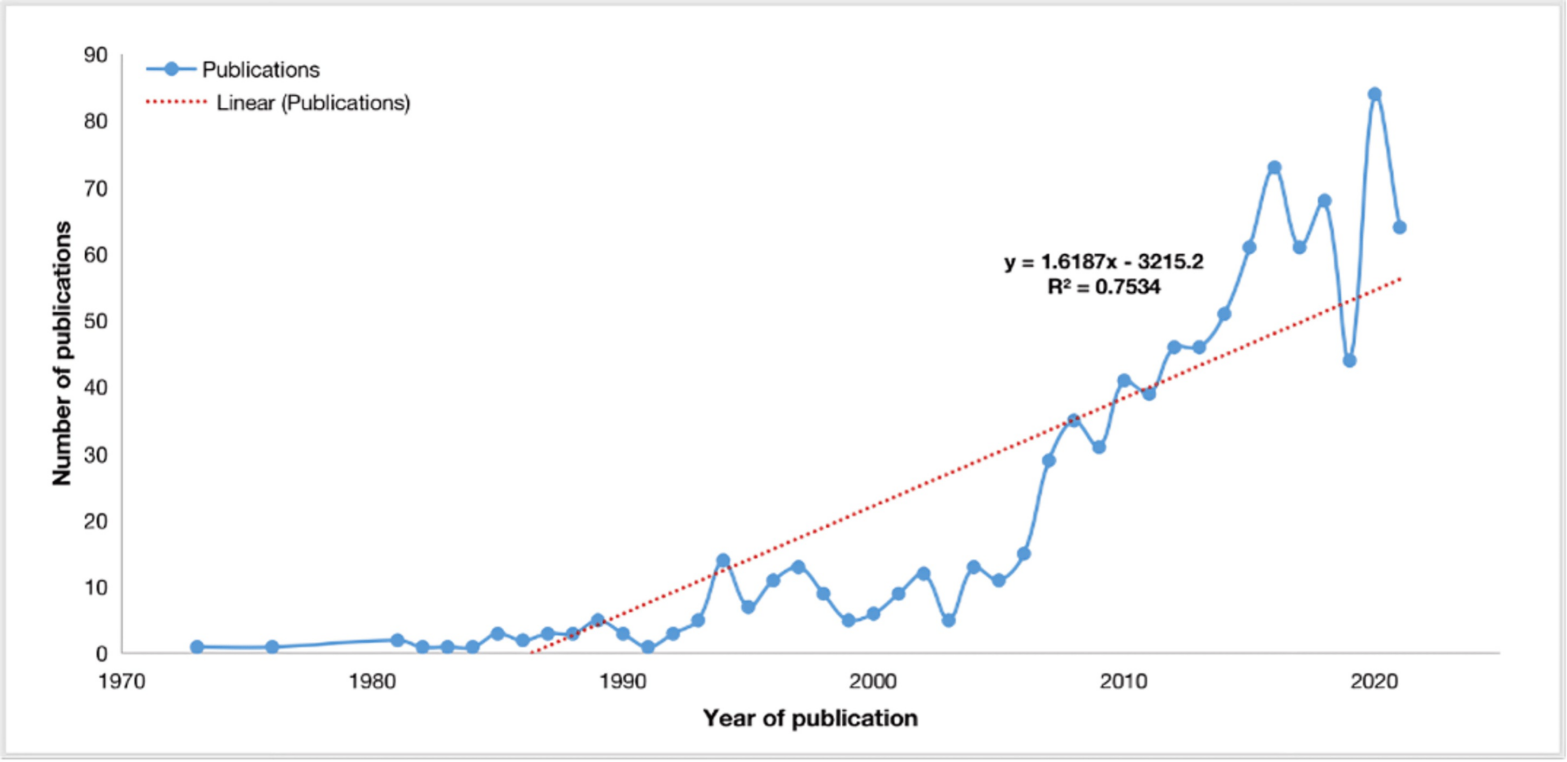
Annual scientific production The image was created by the authors using the Web of Science platform.

The annual scientific production growth rate was found to be 10.41%. The most frequent years of publications were 2020 (n = 84), 2016 (n = 73), and 2018 (n = 68), as shown in Figure 2. It is possible that the impact of COVID-19 may have had an impact on the increase in the production of articles in 2020, as lockdown restrictions imposed may have reduced the accessibility to clinical research.

Most Frequently Published Journals

Journals that published at least 10 studies are presented in Table 2. The most frequently published journal was “Foot and Ankle International” (n = 121), followed by “Journal of Foot and Ankle Surgery” (n = 85) and “Arthroscopy - Journal of Arthroscopic and Related Surgery” (n = 63). However, according to Bradford’s law, which estimates the diminishing returns of searching for references in multiple scientific journals, only four journals were core journals or zone 1 journals; these include “Foot and Ankle International," “Journal of Foot and Ankle Surgery," “Arthroscopy - Journal of Arthroscopic and Related Surgery," and “Knee Surgery Sports Traumatology Arthroscopy."

**Table 2:** Journals with at least 10 publications IF = impact factor

Journal	Number of publications	IF 2021
Foot and Ankle International	121	3.569
Journal of Foot and Ankle Surgery	85	1.345
Arthroscopy - Journal of Arthroscopic and Related Surgery	63	5.973
Knee Surgery Sports Traumatology Arthroscopy	46	4.114
Foot and Ankle Surgery	40	2.84
Arthroscopy Techniques	37	1.47
Clinics in Podiatric Medicine and Surgery	33	0.769
Techniques in Foot and Ankle Surgery	32	0.36
Arthroscopy	21	5.973
Orthopaedics and Traumatology: Surgery and Research	17	2.425
American Journal of Sports Medicine	16	7.01
Archives of Orthopaedic and Trauma Surgery	16	2.928
Clinics in Sports Medicine	15	2.186
Orthopedics	14	1.345
Current Orthopaedic Practice	13	0.217
Journal of Bone and Joint Surgery	12	6.558
Operative Techniques in Sports Medicine	12	0.318
Foot	11	1.317
Foot and Ankle Specialist	11	1.43
Injury	11	2.687
Sports Medicine and Arthroscopy Review	10	2.617

Most Prolific Authors

The most prolific authors are presented in Table 3. The leading author was Lui TH (n = 35), followed by Takao M (n = 25) and Amendola A (n = 20), highlighting their interest and expertise in this area. 

**Table 3:** Most prolific authors

Authors	Number of publications	Articles Fractionalized
Lui TH	35	26.25
Takao M	25	4.57
Amendola A	20	4.80
Vega J	19	4.70
Ferkel RD	18	7.59
Van Dijk CN	15	4.28
Dalmau-Pastor M	14	3.30
Maffulli N	14	3.86
Digiovanni CW	13	2.57
Ochi M	13	2.34
Uchio Y	13	2.52
Malagelada F	12	2.55
Bauer T	10	1.95
Giza E	10	1.94
Guillo S	10	2.16
Karlsson J	10	2.25
Lee JW	10	2.04

Most Relevant Affiliations

The most frequently appeared affiliations were North District Hospital (n = 50), Harvard Medical School (n = 46), and University of Amsterdam (n = 44), as shown in Table 4.

**Table 4:** Most frequently cited affiliations Note: In total, 37 publications did not describe any affiliation and thus were excluded from the above table.

Rank	Affiliations	Country	Number of publications
1	North District Hospital	Hong Kong	50
2	Harvard Medical School	The United States	46
3	University of Amsterdam	The Netherlands	44
4	Hiroshima University	Japan	43
4	Hospital for Special Surgery	The United States	43
6	University of Barcelona	Spain	37
7	Shimane Medical University	Japan	34
8	University of California	The United States	30
9	Duke University Medical Center	The United States	29
9	Hallym University College of Medicine	Korea	29
10	Tokushima University Graduate School	Japan	26
11	Shimane Univ. School of Medicine	Japan	21

Keyword Analysis

**Figure 3:**
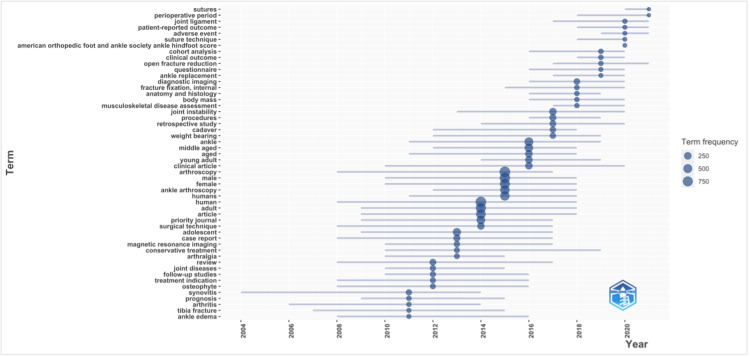
Trend topics between 2011 and 2021 The image was created by the authors using the Web of Science platform.

As shown in Table 5, the most frequently used keywords were "arthroscopy" (n = 932), "human" (n = 917), "male" (n = 850), "adult" (n = 775), "female" (n = 773), "article" (n = 675), "ankle arthroscopy" (n = 614), "humans" (n = 610), "ankle" (n = 468), and "priority journal" (n = 439). This suggests that the focus of research in ankle arthroscopy is in high-quality journals and carried out on patients. The most studied trend topics over the last decade are presented in Figure 3.

**Table 5:** Top 50 most frequently used keywords in the included articles

Keyword	Number of times used (n)
Arthroscopy	932
Human	917
Male	850
Adult	775
Female	773
Article	675
Ankle arthroscopy	614
Humans	610
Ankle	468
Priority journal	439
Middle aged	419
Ankle joint	387
Adolescent	344
Treatment outcome	336
Talus	281
Nuclear magnetic resonance imaging	257
Aged	253
Ankle injuries	242
Young adult	238
Clinical article	235
Joint instability	233
Ankle injury	226
Follow-up	220
Surgical technique	220
Arthroscopic surgery	185
Case report	176
Procedures	174
Debridement	151
Ankle instability	150
Review	149
Retrospective study	144
Controlled study	142
Ankle pain	141
Ankle fracture	134
Retrospective studies	130
Osteoarthritis	128
Major clinical study	126
Diagnostic imaging	123
Postoperative complication	120
Ankle radiography	114
Computer-assisted tomography	112
Cadaver	109
Postoperative period	107
Weight bearing	106
Magnetic resonance imaging	102
Range of motion	101
Tibia	98
Postoperative care	95
Synovitis	93
Osteosynthesis	92

Countries or Territories Involved in Ankle Arthroscopy Research

The largest number of publications was produced in the United States (n = 376), followed by the United Kingdom (n = 107), Japan (n = 68), South Korea (n = 59), and Italy (n = 51), as shown in Table 6. 

**Table 6:** Countries or territories involved in ankle arthroscopy research. A total of 19 publications were excluded from the above table as they had an unknown country or territory of origin.

Country	Number of publications
United States	376
United Kingdom	107
Japan	68
South Korea	59
Italy	51
China	45
Spain	45
Netherlands	38
France	37
Germany	32
Hong Kong	25
Turkey	25
Canada	23
Switzerland	20
Egypt	15
Sweden	13
Australia	12
Austria	10
Brazil	8
Croatia	8
India	8
Poland	8
Portugal	8
Belgium	7
Saudi Arabia	7
Ireland	6
Argentina	4
Chile	4
Greece	4
Singapore	4
Thailand	4
Taiwan	3
Colombia	2
Denmark	2
Hungary	2
Israel	2
Lebanon	2
Malaysia	2
Qatar	2
Andorra	1
Bahrain	1
Czech Republic	1
Grenada	1
Guadeloupe	1
Indonesia	1
Iran	1
Kuwait	1
Martinique	1
New Zealand	1
Oman	1
Philippines	1
Russian Federation	1
South Africa	1
Venezuela	1
Vietnam	1

Institutions' and Countries' Collaboration Network

**Figure 4:**
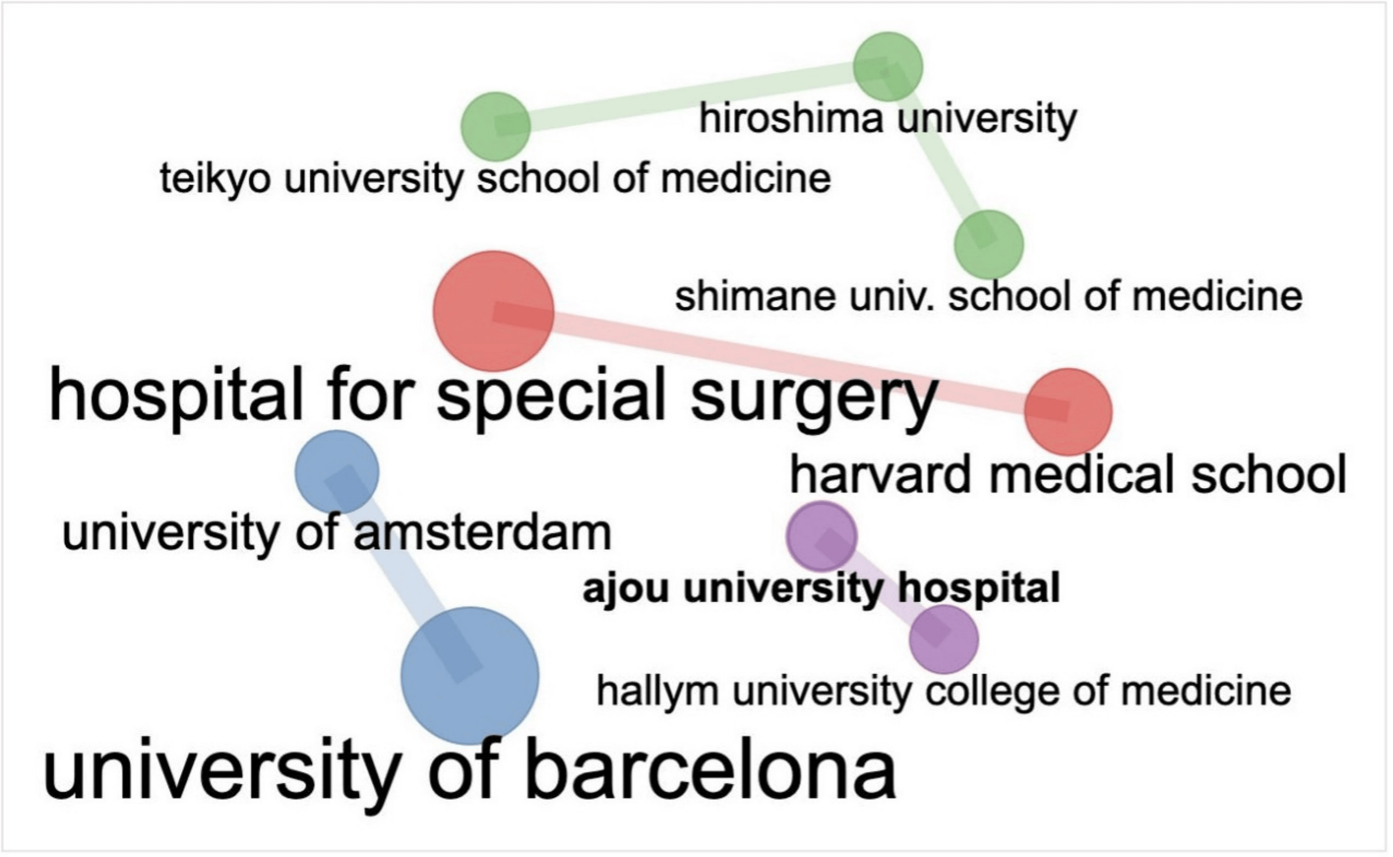
Collaboration network among the most active institutions The image was created by the authors using the Bibliometrix platform.

**Figure 5:**
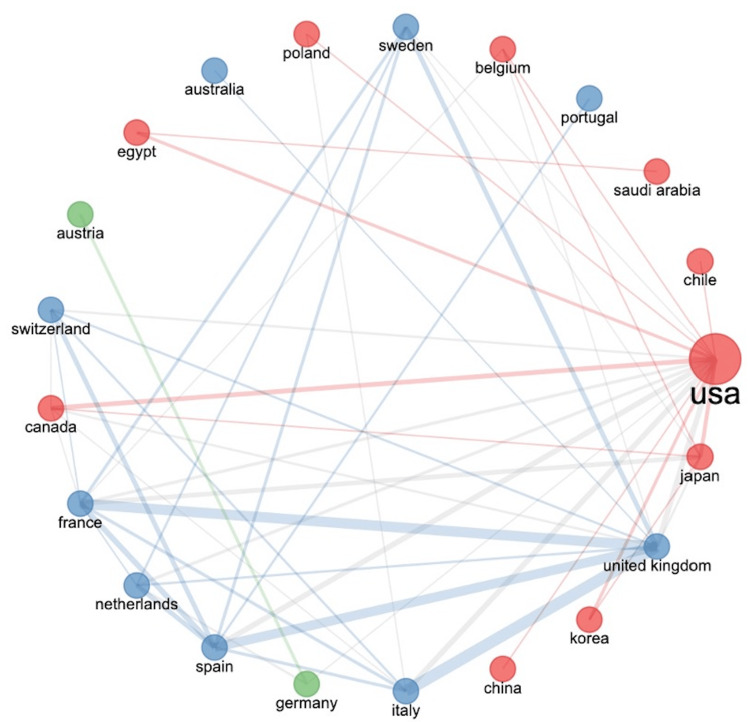
Collaboration network between countries The image was created by the authors using the Bibliometrix platform.

As shown in Figure 4, the University of Barcelona has strongly collaborated with the University of Amsterdam. As shown in Figure 5, the United Kingdom had the strongest collaboration with Italy, France, and Spain, while the United States had the strongest collaboration with Italy, Spain, and the United Kingdom. 

Discussion

Ankle arthroscopy is commonly performed by foot and ankle surgeons worldwide for the evaluation and management of a range of ankle joint pathologies. To the best of the authors' knowledge, this is the first bibliometric visualization analysis on the topic of ankle arthroscopy indexed in the Scopus® database. Bibliometric analyses offer valuable data on the history of scientific publications on a particular topic, in this case, ankle arthroscopy [11].

As can be seen in Table 1, each of the included articles was published between 1973 and 2021. Most of these articles were original articles (n = 778), while a minority were review articles (n = 160). This is not surprising considering that research has only accelerated over the past 20 years (Figure 2), and a requirement for a review article is a significant number of original articles. Interestingly, there was a large number (n = 175) of different journals that published studies on ankle arthroscopy (Table 1). However, three of the top five most frequently published journals on ankle arthroscopy articles were dedicated foot and ankle journals (Table 2). Again, this is unsurprising considering this study is based on ankle arthroscopy.

Figure 2 clearly illustrates the growth in ankle arthroscopy research since the early 2000s. It is likely that this is due, at least in part, to the ever-increasing demand for more accurate surgery, quicker healing times and lower complication rates [12]. In addition, patient demand for arthroscopic and minimally invasive surgery continues to soar [12]. As can be seen in Figure 2, publications on ankle arthroscopy continue to grow, with 2020 seeing the highest number of publications on record (n = 84). It is likely that this trend will continue as the demand for evidence-based treatment modalities increases, in addition to the advancement in current surgical techniques.

The journal with the highest number of publications was "Foot and Ankle International" (n = 121; Table 2). This is not surprising for several reasons. First, "Foot and Ankle International" is the official journal of the American Orthopaedic Foot and Ankle Society and has the highest impact factor (IF 3.569; Table 2) that is specific for foot and ankle research. In addition, the USA had the highest number of publications included in this study (n = 376; Table 6). Finally, this same finding has been observed in other foot and ankle bibliometric analyses [6-8].

Three journals have published more than 50 research papers on ankle arthroscopy ("Foot and Ankle International," "Journal of Foot and Ankle Surgery," and "Journal of Arthroscopic and Related Surgery"; Table 2). Each of these journals is based out of the USA, which highlights the demand for ankle arthroscopy research in the USA. One potential explanation for this is the large number of foot and ankle surgeons in the USA. In the USA, while orthopedic surgeons perform much of the foot and ankle surgery, particularly ankle and hindfoot procedures, podiatrists (Doctors of Podiatric Medicine) also perform foot and ankle surgery. This reflects the variation in training and licensing requirements in the USA. This is not the case in many countries around the world where training and licensing requirements differ, and only orthopedic surgeons may operate. This includes many countries in Europe (with the exception of the UK and a small number of countries in mainland Europe).

The most prolific author was Lui TH, based in the Department of Orthopedics, North District Hospital in Hong Kong, with a large number of publications of 35 (Table 3) and high articles fractionalized of 26.25 (Table 3). This highlights that Lui TH is one of the greatest contributors and most influential authors in terms of the number of publications in the field of ankle arthroscopy. Other significant contributors to the field of ankle arthroscopy were Takao M (n = 25; Table 3) and Amendola A (n = 20; Table 3). No other author contributed to more than 20 publications.

Given that Lui TH was based out of North District Hospital in Hong Kong, it is unsurprising to see that this institution had a greater number of affiliations with published research articles (n = 50; Table 4). Harvard Medical School was closely behind with 46 affiliations (n = 46), while the University of Amsterdam was affiliated with 44 publications (n = 44).

As can be seen in Table 6, a total of 55 countries have published research on ankle arthroscopy. This highlights the global need for high-quality research in ankle arthroscopy and suggests that significant advances in arthroscopic techniques and outcomes are still sought worldwide. Interestingly, the USA has published more than three times the number of articles (n = 307) compared to the second most prolific nation, which was the United Kingdom (n = 107). This was followed by Japan (n = 68), South Korea (n = 59), and Italy (n = 51). This may highlight the significant discrepancy between developed and developing nations in terms of research funding and subsequent research output. Previously published bibliometric studies in different research fields show that the highest number of publications originated from the USA [13-27].

The keyword analysis is represented in Table 5. As was expected, the most frequently used keyword across the publications was "arthroscopy", which was featured in 932 out of 938 articles (99.36%). Other very important keywords for authors were "human" (n = 917), "male" (n = 850), "adult" (n = 775), and "female" (n = 773). This is valuable information for researchers who are conducting a search of the literature on ankle arthroscopy. The most studied trend topics over the past decade are presented in Figure 3. The most focused trend topics over the past five years (2017-2021) were suture, perioperative period, joint ligament, patient-reported outcome, adverse event, suture technique, American Orthopedic Foot and Ankle Society Ankle Hindfoot Score, cohort analysis, clinical outcome, open fracture reduction, questionnaire, ankle replacement, diagnostic imaging, internal fracture fixation, anatomy and histology, body mass, musculoskeletal disease assessment, joint instability, procedures, retrospective study, cadaver, and weight bearing.

Figure 4 depicts the research collaboration between institutions, while Figure 5 represents the research collaboration amongst various countries. It is clear to see in Figure 4 that the University of Barcelona had the strongest collaboration with the University of Amsterdam. This was closely followed by a strong collaboration between the Hospital for Special Surgery and Harvard Medical School and a significant collaboration between Teikyo University School of Medicine, Hiroshima University, and Shimane University School of Medicine. This is perhaps unsurprising considering that these institutions have published a large number of research articles (Table 4). Figure 5 shows that the United Kingdom had the strongest collaboration with Italy, France, and Spain. One potential explanation for this is the geographical proximity of these countries to one another, in addition to similarities in medical and surgical training within these countries. However, geographical proximity does not explain the United States’ strong collaboration with Italy, France, and Spain (Figure 5).

One possible explanation for the strong collaboration among these countries is that, following orthopedic surgical training, most orthopedic surgeons spend a year abroad completing a fellowship in foot and ankle surgery to hone their operating skills and further develop expertise in their field. Therefore, many foot and ankle surgeons who have fully completed training (known as consultant surgeons or attending surgeons) will have developed relationships with surgeons from other countries. This likely plays a role in promoting future research collaborations among these surgeons in different institutions and countries.

It is clear that developed countries have produced the highest number of research articles (Figure 3, Table 6) and achieved the strongest level of collaboration (Figures 4, 5). This may reflect a greater amount of research funding available to researchers and clinicians within these institutions and countries.

Limitations

This bibliometric analysis is a cross-sectional study, and as a result, all articles published after the date of the literature search were not included. Therefore, this study is only a reflection of the research available at the time of the literature search. This is an unavoidable limitation of cross-sectional studies. In addition, due to resource limitations, only studies published in the English language were included, and therefore it is possible that relevant studies published in a language other than English were excluded. 

## Conclusions

This study provides key developments, trend topics, and collaboration networks in ankle arthroscopy research over the years. The most prolific author, most active institution, and country were Lui TH, North District Hospital, and the United States of America, respectively. The most studied trend topics were arthroscopy, human, male, adult, and female. The largest portion of the included publications was produced in developed countries. The United Kingdom had the strongest collaboration with Italy, France, and Spain. However, the USA had the strongest collaboration with Italy, Spain, and the United Kingdom. The zone 1 or core journals in ankle arthroscopy were “Foot and Ankle International," “Journal of Foot and Ankle Surgery," “Arthroscopy - Journal of Arthroscopic and Related Surgery,” and “Knee Surgery Sports Traumatology Arthroscopy." Future research may identify emerging trends in ankle arthroscopy, identify specific arthroscopic procedures of interest, and include studies published in a language other than English.
